# Pupillary fluctuation amplitude preceding target presentation is linked to the variable foreperiod effect on reaction time in Psychomotor Vigilance Tasks

**DOI:** 10.1371/journal.pone.0276205

**Published:** 2022-10-20

**Authors:** Jumpei Yamashita, Hiroki Terashima, Makoto Yoneya, Kazushi Maruya, Haruo Oishi, Takatsune Kumada

**Affiliations:** 1 Access Operations Project, NTT Access Network Service Systems Laboratories, Nippon Telegraph and Telephone Corporation, Kanagawa, Japan; 2 Department of Intelligence Science and Technology, Graduate School of Informatics, Kyoto University, Kyoto, Japan; 3 Human Information Science Laboratory, NTT Communication Science Laboratories, Nippon Telegraph and Telephone Corporation, Kanagawa, Japan; Julius-Maximilians-Universität Würzburg, GERMANY

## Abstract

Understanding temporally attention fluctuations can benefit scientific knowledge and real-life applications. Temporal attention studies have typically used the reaction time (RT), which can be measured only after a target presentation, as an index of attention level. We have proposed the Micro-Pupillary Unrest Index (M-PUI) based on pupillary fluctuation amplitude to estimate RT before the target presentation. However, the kind of temporal attention effects that the M-PUI reflects remains unclear. We examined if the M-PUI shows two types of temporal attention effects initially reported for RTs in the variable foreperiod tasks: the variable foreperiod effect (FP effect) and the sequential effect (SE effect). The FP effect refers to a decrease in the RT due to an increase in the foreperiod of the current trial, whereas the SE effect refers to an increase in the RT in the early part of the foreperiod of the current trial due to an increase in the foreperiod of the previous trial. We used a simple reaction task with the medium-term variable foreperiods (Psychomotor Vigilance Task) and found that the M-PUI primarily reflects the FP effect. Inter-individual analyses showed that the FP effect on the M-PUI, unlike other eye movement indices, is correlated with the FP effect on RT. These results suggest that the M-PUI is a potentially powerful tool for investigating temporal attention fluctuations for a partly unpredictable target.

## Introduction

Raising expectations about future behavioral demands, known as “temporal attention” (also known as “temporal expectations” or “temporal preparations”), is ubiquitously employed in a broad range of human behavioral control tasks [1]. Temporal attention is essential for time-accurate control of behavior. For example, sprinters need to start moving instantly by adjusting their attention to the timing of the starting pistol’s sound. Drivers prepare their attention to start, stop, lane change, gas pedal, brakes, and steering wheel operations by observing the traffic situation. Moreover, we often implicitly engage temporal attention even in seemingly static situations [2–4]. For example, we expect a web page to load successfully immediately after opening the page when browsing the Internet. We also expect an error message if the page is not displayed after some time [3]. Users prepare for their subsequent behavior based on these expectations. Therefore, clarifying temporal attention mechanisms will help us understand our everyday cognitions and assist in developing user-friendly human-computer interactions (HCI) [5,6].

**Fig 1:**
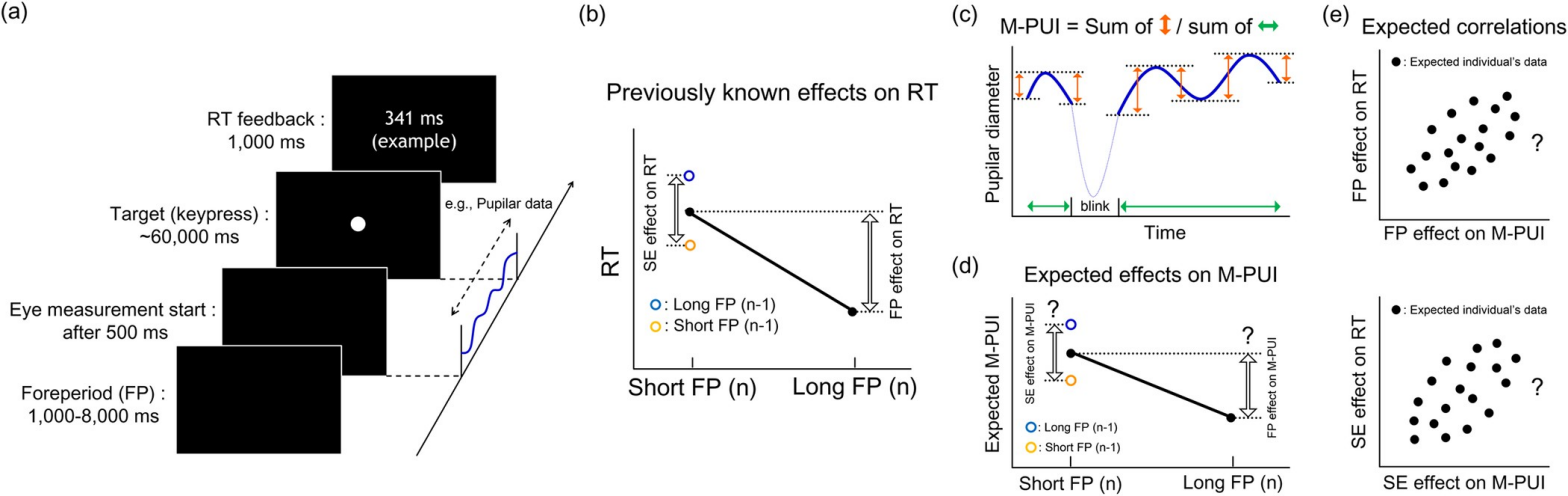
Summary of current study’s procedure and hypothesis. **(a) Serial flow of elements in one trial of the Psychomotor Vigilance Task.** A white dot target appeared after a random interval ranging from 1,000 to 8,000 ms in 250 ms increments. We asked the participants to make a keypress for the target appearance as quickly as possible. After the response, the feedback of the RT was displayed. We used the pupilar diameter from 500 ms after the start of the trial to the target appearance to exclude the possible noise (cf. [24]). **(b) Previously known temporal attention effects on RT, i.e., FP/SE effects on RT.** RT should be shorter when FP (n) is longer than when FP (n) is shorter. The difference in RT between short and long FP (n) is defined as the FP effect on RT (cf. [24]). As for the SE effect, RT should be longer when FP (n-1) is longer than when FP (n-1) is shorter only for the short FP (n). The difference in RT between long FP (n-1) and short FP (n-1) is defined as the SE effect on RT (cf. [24]). **(c) The conceptual example of M-PUI calculation.** The solid blue line represents the valid smoothed pupilar diameter. The dotted blue line represents the invalid smoothed pupilar diameter during a blink. We first calculated the absolute value of change in pupillary fluctuation degree (sum of the magnitude of orange arrows). Then, the absolute degree of change was divided by the length of a valid pupilar diameter (sum of the magnitude of green arrows). **(d) The expected temporal attention effects on M-PUI; i.e., the FP/SE effects on M-PUI.** Similar to RT, the FP/SE effects on M-PUI can be expected, but it has not been investigated to date. **(e) The expected inter-individual positive correlation between temporal attention effects, i.e., the FP/SE effects on RT and M-PUI.** If the FP/SE effects on M-PUI are positively correlated inter-individually with those of RT, it will provide further evidence that M-PUI reflects temporal attention in the variable FP task.

Researchers have investigated temporal attention using the interval periods, i.e., “foreperiods (FPs) [7,8].” Typically these experimental paradigms present a signal announcing the start of a trial (i.e., a warning signal), an FP after the warning signal, and finally, a target requiring the participants to react as fast as possible [7,8]. A shorter reaction time (i.e., RT) to the target is interpreted as higher temporal attention formed in the FP. Such experiments can be divided into two major categories: those in which the FP is fixed within a block (fixed FP) and those in which FP is variable within a block (variable FP) [8]. A general upperward slope [7,8], or a U-shaped curve [9], is typically observed in graphs plotting the RT for each fixed FP. The RT is the shortest when the fixed FP is relatively short, and increases as the fixed FP is shortest or gets longer. That is, temporal attention in the fixed FP paradigm might be maximally enhanced at a predictable target presentation time, and this attentional enhancement might be easiest at a certain short FP and become harder at longer FP due to the reduced time estimation accuracy [8]. On the other hand, more complex phenomena are observed in a variable FP-RT graph (cf. Fig 1(B)). In this graph, the FP of the current trial is FP (n), and the FP of the previous trial is FP (n-1). In the variable FP paradigm, a downward slope is observed for the FP (n)-RT function, i.e., the variable foreperiod effect (FP effect) [8,10]. More precisely, the downward slope in FP (n)-RT is steeper for longer FP (n-1) but shallower for shorter FP (n-1), indicating differences due to FP (n-1) when FP (n) is short, but almost no differences due to FP (n-1) when FP (n) is long; i.e., the Sequential effect (SE effect) [10]. Several psychological models have attempted to explain the FP and SE effects [11–15]. Although it is still debated as to which models are valid, it seems that temporal attention might be low at short FP (n) but increase at longer FP (n) according to the target presentation distributions. The variable FP paradigms can be further divided in terms of the range of FPs. These paradigms include typical temporal attention studies using variable FP of less than one second as the shortest and approximately two seconds as the most extended (the short-term variable FP paradigm) [16–18]. Moreover, some recent studies have used the Psychomotor Vigilance Tasks (PVT) [19], often with variable FP ranging from about 2 to 10 seconds (the medium-term variable FP paradigm) [20–24]. The medium-term FP studies using PVT with many different FPs often combine several ranges of FPs into fewer groups [20–24].

A significant challenge for previous research on temporal attention in the fixed and variable FP tasks has been the need for a specific response to target presentations because detecting how much temporal attention a participant has developed during the FP before the target presentation is impossible. Estimating the users’ temporal attention during the FP in controlled experiments would be the first step in developing user-friendly systems that analyze users’ temporal attention and developing advanced adaptive interfaces that adjust the information presentation timing by sensing users’ temporal attention.

The pupilar diameter can be used to estimate the degree of temporal attention robustly in fixed FP and partially in medium-term variable FP tasks. The muscles that influence the pupilar diameter, which is innervated by the autonomic nervous system, reflect the cortical state of the central nervous system related to temporal attention [24–34]. As shown in Table 1, pupillometry studies [24–26] can be approximately classified into four types based on the combination of two FP types (fixed or medium-term variable) and two methods of examining pupilar diameter (pre- or post-target presentation).

**Table 1:** Summary of previous pupillometry studies on temporal attention.

		Fixed FP	Medium-term variable FP	
		Temporal effect	FP effect	SE effect
Pupillary indices after target presentation	Post-target transient pupilar dilation	• (Not examined)	**Larger for shorter FP (n), particularly with reward [24]**	• No difference for FP (n-1) [24]
**Pupillary indices before or at target presentation**	Pre-target average pupilar size	**Increases at the time of target presentation [25, 26]**	• No consistent difference for FP (n) [24]	• No difference for FP (n-1) [24]
	Pupillary fluctuation amplitude (M-PUI)	• (Not examined)	• **Not examined but expected to be different by the trial-by-trial correlation with RT [35]**	• **Not examined but expected to be different by the trial-by-trial correlation with RT [35]**

The current study focused on estimating temporal attention in the medium-term variable FP using the pre-target pupilar index among these four categories. In reality, it is unlikely that users always know the timing of the target presentation. Therefore, estimating temporal attention to partly unpredictable targets (i.e., targets after variable FPs) is particularly necessary. To our knowledge, there are no previous pupillometric studies of the short-term variable FP paradigm, although there are studies of the medium-term variable FP paradigm. If we were to develop systems that interact with users based on their temporal attention estimates, it would be desirable to target medium-term temporal attention situations, including driving (excluding urgent reactions) and computer work situations, rather than short-term temporal attention situations such as a sprinter waiting for the sound of a pistol. Also, such estimates must be conducted before presenting the target. Therefore, the estimation must be conducted using the pre-target pupilar index. Several findings suggest that the pre-target pupilar index might reflect temporal attention in fixed FP tasks [25,26]. The post-target transient pupilar dilation partly reflects differences in the FP (n) [24], which might include the degree of temporal attention in medium-term variable FP tasks. However, unfortunately, no pupilar index has been developed to reflect temporal attention before target presentation when the medium-term FP is variable.

A better index for estimating temporal attention in the medium-term variable FP is the pupillary fluctuation amplitude before target presentation, or the Micro-Pupillary Unrest Index (M-PUI) [35]. We have recently proposed the M-PUI, a quantitative index of pupilar diameter fluctuation degree over a shorter timescale [35], which was inspired by the better-known “Pupillary Unrest Index (PUI)” of pupillary unrest over a longer timescale [36]. In our previous paper, we reported a within-individual trial-by-trial positive correlation between M-PUI and RT (i.e., the M-PUI’s reflection of short-term vigilance levels) using the PVT with the medium-term variable FP [35]. That is, we found that a participant’s M-PUI in long RT trials was significantly larger than that in short RT trials without considering the FP length. However, from the perspective of medium-term variable FP studies, this trial-by-trial positive correlation could have been mediated by both FP and SE effects, only the FP effect, only the SE effect, or none of these effects [35]. Notably, the positive correlation disappeared when the M-PUI calculation period was more than a few seconds away from the time immediately before the target presentation, suggesting that the real-time M-PUI reflects RT with a temporal resolution of a few seconds. This temporal resolution implies that the M-PUI might have reflected the degree of second-to-second increase in temporal attention during the range of FPs between a minimum of one second and a maximum of eight seconds in our PVT. Therefore, the M-PUI might decrease as temporal attention increases during the FP (n) (i.e., the RT decreases during the FP (n), or the FP effect) depending on its initial level determined by the FP (n-1) (i.e., the RT in the short FP (n) depending on the FP (n-1), or the SE effect: cf. Table 1). Similarly, we suggested the theoretical possibility that M-PUI might capture the locus-coeruleus norepinephrine (LC-NE) system’s preparatory state for the target detection’s phasic enhancement [35]. These previous findings and theoretical possibilities suggest that M-PUI might reflect temporal attention in preparation for a target, i.e., the FP or SE effect [35]. Nevertheless, we did not test this hypothesis but merely proposed the possibility that positive correlations are mediated by the FP and SE effects due to the limited scope of the previous study.

The current study, in contrast, investigated the relationship between the M-PUI and RT differences in different FPs (n/n-1) to examine the possibility that M-PUI reflects the effects of FP and SE on RT (cf. Fig 1). As described in Fig 1(A), the participants were required to respond to a target presented after variable FPs. We examined differences in the RT/M-PUI between short and long FPs (see the procedure of [24]) that might be caused by temporal attention differences (e.g., [8,10]) to ensure a sufficient number of trials for calculating the indices. As seen in Fig 1(B), previous studies [8,10] have suggested two types of temporal attention effects on RT in medium-term variable FP tasks. These effects include the primary effect on RT, or the FP effect, which is the RT for short FP (n) minus that for long FP (n) (cf. [24]), and the secondary effect, or the SE effect, which is the RT for short FP (n) after long FP (n-1) minus that after short FP (n-1) (cf. [24]). As seen in Fig 1(C), we calculated M-PUI, a measure of pupillary fluctuation amplitude for each FP. We examined whether the FP/SE effects on M-PUI were similar to RT. See Fig 1(D). If the M-PUI shows similar patterns to RT in terms of the FP/SE effects, it would suggest that M-PUI reflects dynamically changing temporal attention in the medium-term variable FP tasks. We also examined the inter-individual positive correlation between the FP/SE effects on RT and M-PUI, See Fig 1(E). If the FP/SE effects on M-PUI reflected individual differences in RT (cf. [21,24,37,38]), it would prove that M-PUI robustly reflects temporal attention in medium-term variable FP tasks.

Finally, we confirmed that other eye movement indices did not substantially mediate inter-individual correlations between the FP/SE effects on RT and M-PUI. For example, the pupilar diameter measured from a camera placed facing the face varies depending on the gaze position [39]. We examined the mean pupilar diameter, eye-opening time, number of eyeblinks, or gaze position variations. If no other eye movement indices correlated inter-individually with the RT as strongly as M-PUI, it would be evidence that pupillary fluctuation amplitude is a particularly efficient indication of temporal attention.

To test these hypotheses, we used the data we collected in the previous study [35] because we collected the data to test the hypotheses on estimating general attentional states. Before reusing the previous study’s data in the current study, we assessed the appropriateness of reusing data from two perspectives: the nature of the task and statistical tests. As a result of this assessment, we decided that it was appropriate to reuse previously collected data in the current study. The current study mainly examined inter-individual trial-by-trial RT differences (i.e., individual differences in the FP and SE effects), which the previous study [35] had excluded because we normalized the data to analyze within- (intra-) individual trial-by-trial RT differences. In the Discussion, we have discussed other limitations caused by reusing data as a limitation of the current study.

## Experiment

We decided to examine the validity of M-PUI on smoothed pupilar diameter with a 75 ms window during the few seconds before target presentation [35] using a simple reaction task with the medium-term variable FPs [40] (i.e., PVT [19]). Although the PVT was initially developed to measure vigilance (sustained attention) [19], it has been recently used for investigating temporal attention formed in the variable FP [20–24]. Indeed, it has been demonstrated that temporal attention effects of the PVT, i.e., the FP and SE effects, appear in RTs because of the nature of the variable FP paradigm [20–24]. We decided to conduct the current study in the context of the previous studies using PVT [20–24]. The data set on the FP that we had collected included those between 1–8 seconds an intermediate value between the most common PVT with FPs of 2–10 seconds [19] and PVT-B, a popular shortened version, with FPs of 1–4 seconds [41]. Moreover, we used the approximate uniform distribution for generating FPs following the standard PVT procedure. Therefore, we concluded that the previously collected data was appropriate for use in the current study in terms of the nature of the task. Fig 1(A) shows that participants in our PVT responded to an always presented target at a variable interval by pressing a button as quickly as possible.

The Micro-Pupillary Unrest Index (M-PUI) has been proposed as an index of pupillary fluctuation amplitude that can be calculated quickly [35]. As indicated in Fig 1(C), the amplitude pupilar diameter fluctuations in the M-PUI are calculated by adding absolute dilations and constrictions. The M-PUI index of up to 1,000 ms before target presentation was effectively and positively correlated with RT on a trial-by-trial basis. The frequency of pupillary fluctuation amplitude captured by the M-PUI depends on the temporal resolution of smoothed pupilar diameter time series data before calculating the index. In a previous study, the time series of pupilar diameter smoothed with a window size of 100–50 ms, i.e., pupillary fluctuations of 10–20 Hz temporal measurement frequency were effectively correlated with RT on a trial-by-trial basis. We examined RT/M-PUI differences between short and long FP. Previous FP studies using PVT might have combined several FPs ranges into fewer groups in the analysis, such as two [21,23,24], three [20,21], or seven groups [22] because it is difficult to ensure a sufficient number of trials to calculate the indices for each FP in the PVT with different FPs. The number of trials tends to decrease further in pupillometric studies because the pupilar diameter index can be missing due to events such as blinking, resulting in a dichotomous, two-group approach [24]. We grouped short and long FPs for calculating the indices following previous studies [21,23,24]. Then, we used the difference between long/short FPs as a simplified but robust index of the FP-RT (FP-M-PUI) slope [21,24].

### Method

#### Participants

The participants were individuals with normal or corrected-to-normal vision who applied to participate as a part-time job (N = 20, 15 women, age range 20–43 years). They were recruited outside the laboratory and received payment for participating in the study. The recruitment process and the experimental procedures were approved by the NTT Communication Science Laboratory Research Ethics Committee (Reference Number H29-004). All the experiments were conducted according to the 1964 Declaration of Helsinki. Written informed consent was obtained from all individuals that participated in the study.

#### Apparatus and stimuli

Participants sat at a distance of 60 cm from an LCD monitor (144 Hz, 27 inches, 1920 x 1080 pixels). Stimuli were presented using Psychopy2 on a black background. The target was a white dot in the center of the screen, shown at a visual angle of 2.9 degrees. Left eye movements were recorded using the SR Research Eyelink 1000 with a sampling rate of 1,000 Hz. We stabilized the participants’ heads with a chinrest during the entire task.

#### Procedure and design

The participants conducted eight tasks. We used the data of one of these tasks in the current study. The order of the tasks, which took approximately ten minutes to conduct, was randomized. A ten-minute break followed the tasks. Two participants were paired in the task sessions so that one could take a break while the other performed the task. The total procedure took 180–210 minutes, including the time for preparing the apparatus.

The PVT took approximately 10 min. Fig 1(A) shows that the participants performed the PVT after the eye tracker was calibrated. They were instructed to respond to a target at a variable FP (equally distributed between 1,000 to 8,000 ms in 250 ms increments) by pressing the “space” key of the keyboard as quickly as possible. The disappearance of instructions for the first trial and feedback in the subsequent trials, i.e., the entire screen going dark, was the warning signal for starting a new trial, and the participants were instructed to this effect. The FP was computed from this warning signal. We selected a uniform distribution because it is the standard method of generating FPs in PVT. We adopted an approximation procedure in which the participants were randomly presented with 29 lengths of FPs×4 trials. The fixation point was not presented during the FP to exclude any unwanted pupilar effects caused by the presence of light (cf. [42]). The RT was displayed for 1,000 ms in response to a keypress. The message “False Alarm!” was displayed if a response was made before the target presentation, and the message “Miss!” was displayed if a response was not made within 60 seconds (cf. [19]). The subsequent trial was started after the presentation of each message.

#### M-PUI calculation

We used the pupilar diameter during the interval of 500 ms after starting the trial to target appearance because the pupilar diameter immediately after the start of a trial could include its fluctuations to the target detection/response in the previous trial [24]. We also excluded the time series of the pupilar diameter when the eye was closed before the calculation. Blink detection was conducted by assuming that a blink had occurred when the pupilar diameter’s constant value fell below the threshold and continued to fall until it exceeded that threshold. The threshold was empirically determined as the mean pupilar diameter of the trial × 0.5 to account for individual differences in pupilar diameter’s baseline. The blinking time and 200 ms before and after blinks that could cause artifacts were excluded (cf. [35]).

The procedure for calculating the M-PUI was adopted from our previous study [35]. Firstly, we detected blinks using the above rule. Secondly, we linearly interpolated the pupil diameter’s time series during a blink. Thirdly, we smoothed the pupilar diameter’s time series noise by calculating moving averages by moving a Hanning window of 75 ms in the raw pupilar diameter’s time series. Fourthly, we calculated the degree of change in pupillary fluctuations’ absolute value (the sum of the magnitudes of orange arrows). Finally, we divided the absolute value of the degree of change by the length of specific, valid intervals (sum of the magnitudes of the green arrows).

#### Calculation of eye movement indices

We calculated the mean pupilar dilation, standard deviation (SD) of the pupilar diameter, mean eye closure time, the mean number of blinks, mean horizontal (i.e., X-axis), and vertical (i.e., Y-axis) distance of the gaze position from the center of the screen, and the SD of the gaze position (X, Y). Specifically, we calculated the mean pupilar dilation for each trial by subtracting the baseline pupilar diameter at the time of starting the interval (i.e., 500 ms after starting the trial) from the pupilar diameter at a specified interval because M-PUI might be correlated with pupilar size (cf. [43]). The standard deviation (SD) of the pupilar diameter was calculated because the SD, defined to capture the time-series variation of pupilar diameter, might be sufficient to capture the signal. We also calculated the mean eye closure time and the mean number of blinks because of possible artifacts occurring before and after blinks. The mean gaze position distance from the center of the screen and the SD of the gaze position was calculated because the measured pupilar diameter is affected by the gaze position [39]. The index values in the specified intervals for each trial were calculated for all eye movement indices. The means of each condition were calculated for each participant.

### Results

The data of 17 participants were valid. We excluded 3 participants due to technical errors such as inconsistent calibration results. We conducted 1,972 trials, to most of which the participants responded correctly without missing any trials. There were only nine false alarm trials with RTs of 150 ms or less. When calculating the RT, we excluded trials with misses and false alarms (including anticipatory responses shorter than 150 ms). In calculating eye movement indices, including the M-PUI, we excluded trials in which the measured eye was not open for an interval of over 500 ms, i.e., over half of the M-PUI calculation interval of 1,000 ms.

We first plotted M-PUI and RT of second-to-second FPs (n/n-1) to describe the data generally. The FP (n/n-1) of the 1,500 ms point includes trials with FPs between 1,000 to 1,750 ms, the 2,500 ms point includes FPs between 2,000 to 2,750 ms, the 3,500 ms point includes FPs between 3,000 to 3,750 ms, the 4,500 ms point includes FPs between 4,000 to 4,750 ms, the 5,500 ms point includes FPs between 5,000 to 5,750 ms, the 6,500 ms point includes FPs between 6,000 to 6,750 ms, and the 7,500 ms point includes FPs between 7,000 to 8,000 ms FPs. To mitigate the trial size reduction, we changed the trial exclusion criteria to the criteria that the eye was unopened for over 333 ms, i.e., over one-third of the M-PUI calculation interval of 1,000 ms, presented as preliminary analyses because the number of trials per plot was small.

**Fig 2:**
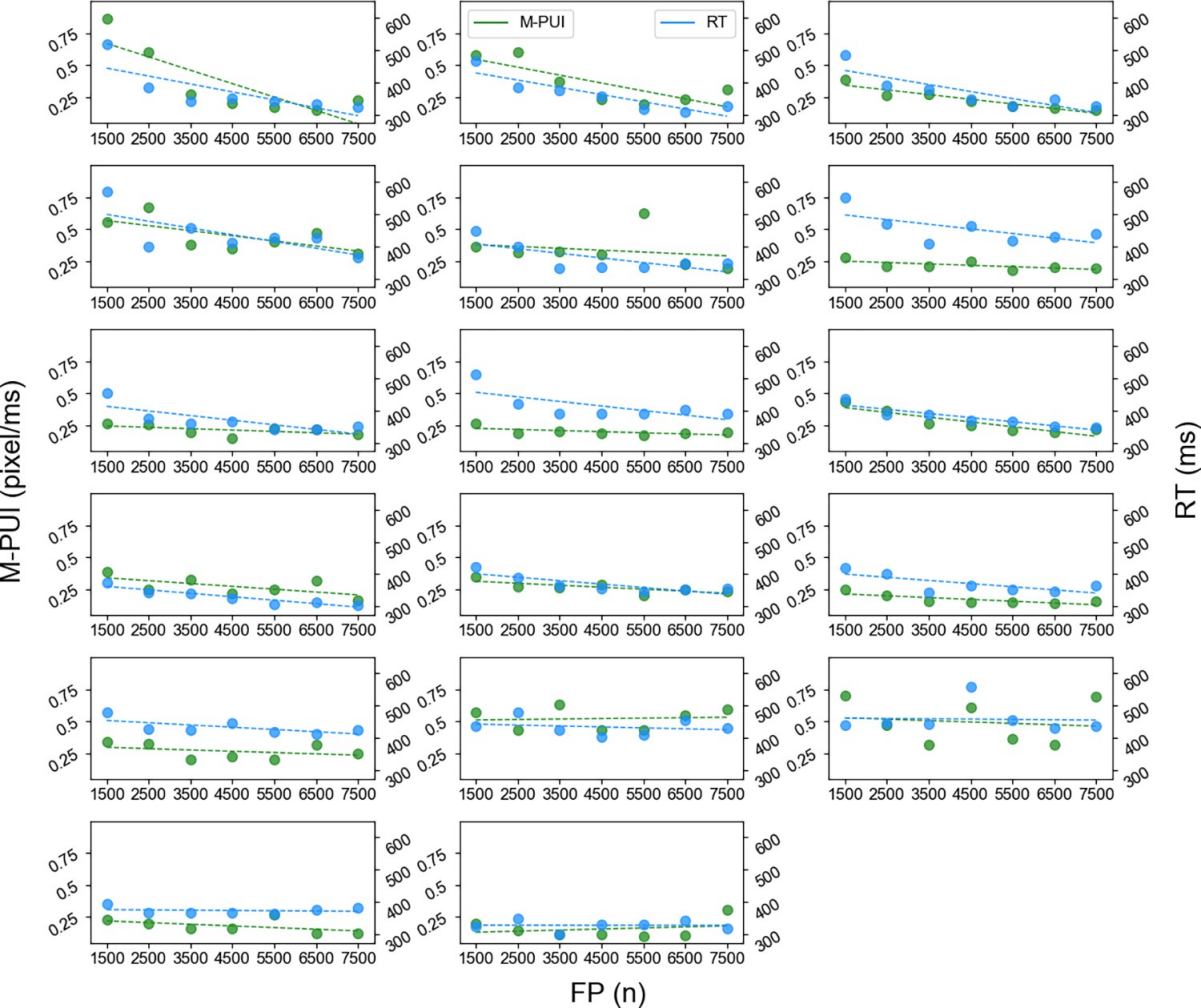
RT and M-PUI for each FP (n).

Regarding the FP effect, the plot for each FP (n) is shown in Fig 2. The FP (n)-RT slope and the FP (n)-M-PUI slope was downward. In Fig 2, both the FP (n)-RT and the FP (n)-M-PUI slopes are plotted in the order from individuals with the steepest FP (n)-RT slope. The FP (n)-M-PUI slope also tended to appear first for individuals with the steepest slope. The Pearson’s correlation coefficient results between the FP (n)-RT and FP (n)-M-PUI slopes indicated strong positive correlations on an individual basis (*r* = 0.77, *p* < .001). Except for the SD of pupilar diameter (*r* = 0.57, *p* < .05), no other eye movement indicators had a significant relationship with RT in terms of FP (n) slopes. The mean number of trials for each plot point was 14.00 (±1.29).

**Fig 3:**
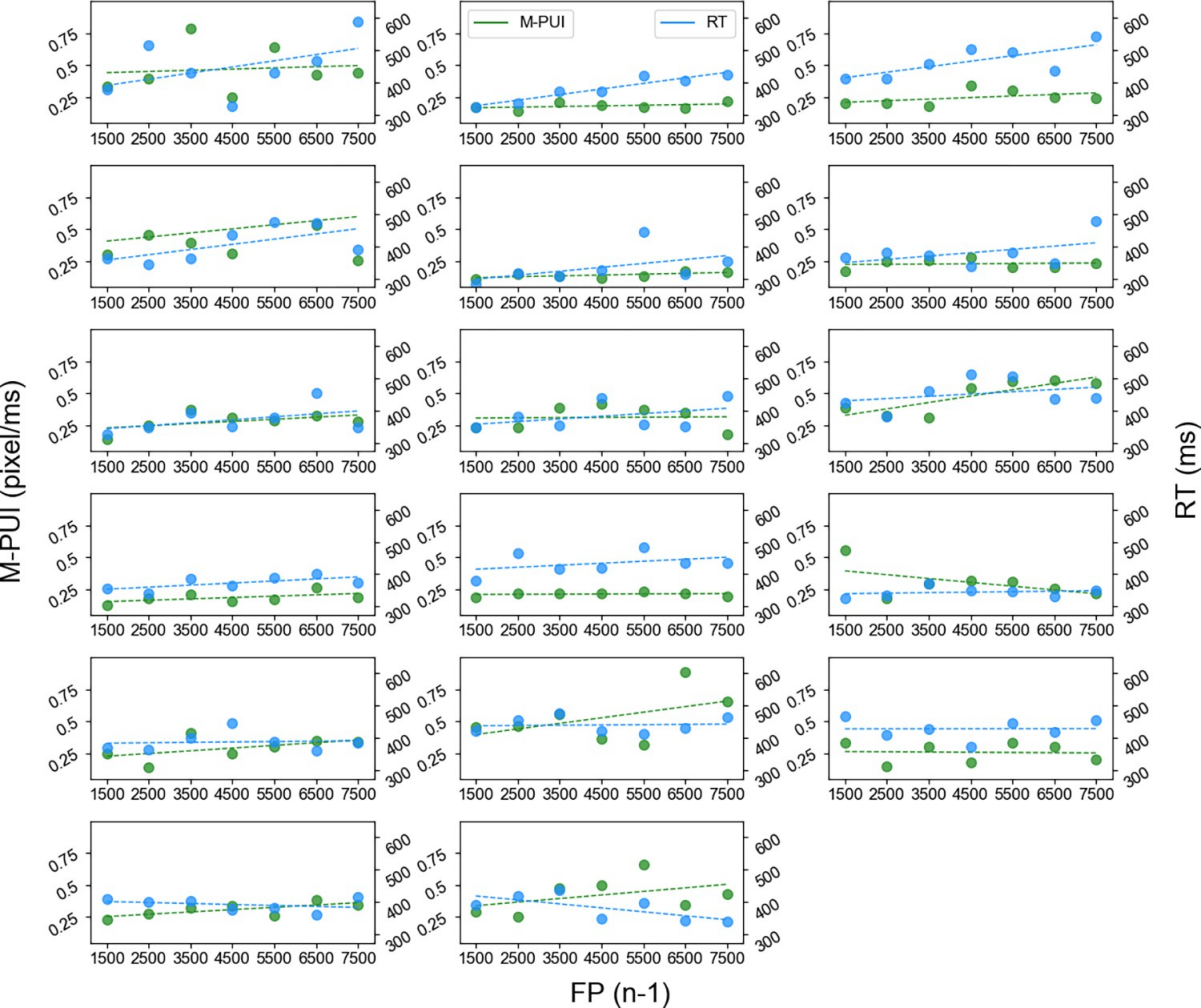
RT and M-PUI for each FP (n-1).

Fig 3 shows the plot of the SE effect for each FP (n-1). Only the trials having an FP (n) shorter than 4,500 ms were included in the analysis. It can be seen that only the FP (n-1)-RT slope moves uppperward. Fig 3 plots FP (n-1)-RT and FP (n-1)-M-PUI slopes in the order from individuals with the steepest FP (n-1)-RT slope, but the FP (n-1)-M-PUI slope varies for each individual. The Pearson’s correlation coefficient between the FP (n-1)-RT and FP (n-1)-M-PUI slopes was nearly zero (*r* = - 0.11, *n*.*s*.). The FP (n-1) slopes showed no other eye movement indicators having a significant relationship with RT except the number of blinks (*r* = 0.65, *p* < .01). The mean number of trials for each plot point was 6.24 (±0.87).

**Fig 4:**
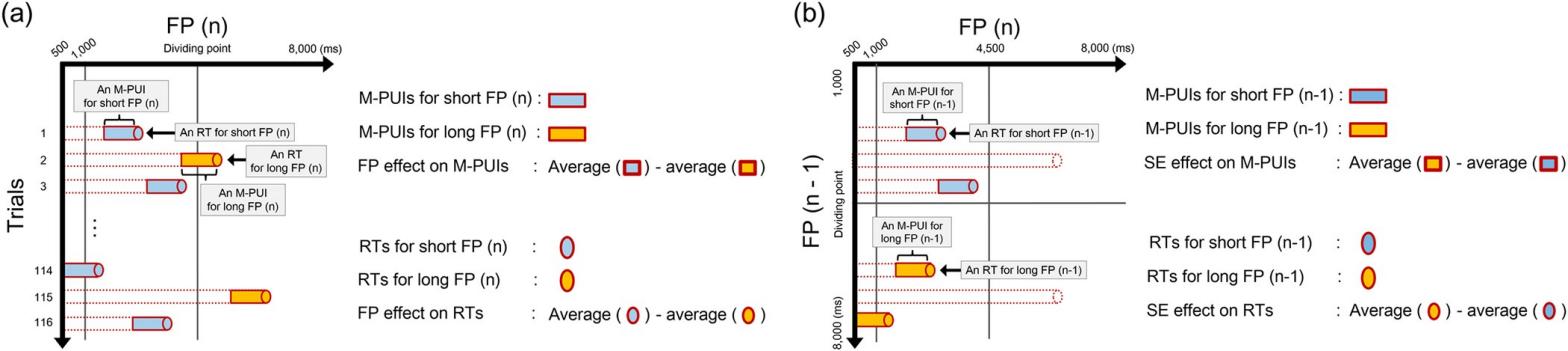
(a) Calculating FP effect on RT and M-PUI, and (b) calculating SE effect on RT and M-PUI.

We examined whether the general pattern of mean M-PUIs for short and long FP (n/n-1), was similar to mean RTs. See Fig 1(B) and 1(D). As shown in Fig 4(A), we first depicted the general FP effect on RTs and M-PUIs. We selected 4,500 ms, the midpoint of the range of FPs, as the dividing point. For each participant, we calculated the mean RTs for trials in which the target appeared at FP (n) for a shorter or an equal time to the dividing point of 4,500 ms; i.e., the mean RTs for short FP (n), and those for trials in which the target appeared at FP (n) for longer than 4,500 ms; i.e., the mean RTs for long FP (n). For each participant, we also calculated the mean M-PUIs from 1,000 ms before target presentation to the target presentation for trials in which the target appeared at FP (n) for a shorter or an equal time to 4,500 ms; i.e., mean M-PUIs for short FP (n), and those for the trials in which the target appeared at FP (n) for longer than 4,500 ms; i.e., mean M-PUIs for long FP (n).

**Fig 5:**
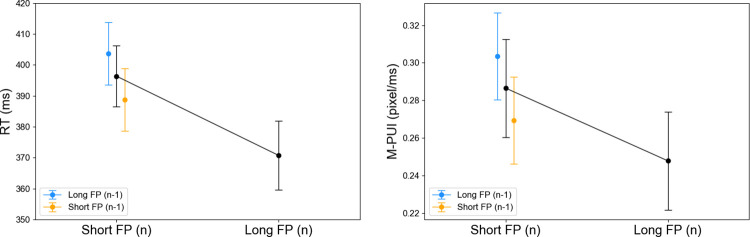
General FP/SE effects on RT and M-PUI. Error bars represent standard errors.

The mean RTs/M-PUIs for short FP (n) and long FP (n) are shown in Fig 5. Seventeen participants are acceptable for t-tests in medium-term variable FP studies [20] because this sample size range is used for basic tests of the FP effect between approximately two conditions. As expected, a one-tailed paired-samples t-test revealed that the mean RTs for short FP (n) were significantly larger than those for long FP (n), (*t*(16) = 4.80, *d* = 0.59, *p* < .0001) and the mean M-PUIs for short FP (n) was significantly larger than those for long FP (n), (*t*(16) = 2.31, *d* = 0.36, *p* < .05). The mean numbers of trials for short and long FP (n) were 38.06 (±8.40) and 45.53 (±7.23), separately.

As shown in Fig 4(B), we depicted the general SE effect on RTs and M-PUIs. For each participant, we calculated the mean RTs for trials in which the target appeared at FP (n) for shorter than 4,500 ms after a trial in which the target appeared at FP (n-1) for shorter than or equal to the dividing point of 4,500 ms; i.e., mean RTs for short FP (n-1), and those after a trial in which the target appeared at FP (n-1) for longer than 4,500 ms; i.e., mean RTs for long FP (n-1). For each participant, we also calculated the mean M-PUIs from 1,000 ms before the target presentation to the target presentation in trials with FP (n) shorter than 4,500 ms after a trial with FP (n-1) longer than 4,500 ms; i.e., mean M-PUIs for long FP (n-1), and those after a trial with FP (n-1) shorter than or equal to 4,500 ms; i.e., the mean M-PUIs for short FP (n-1). The mean RTs and M-PUIs for short FP (n-1) and long FP (n-1) are shown in Fig 5. Although one-tailed paired-samples t-test revealed that the average RTs for long FP (n-1) were significantly larger than those for short FP (n-1), (*t*(16) = 1.77, *d* = 0.36, *p* = .05), the average M-PUIs for long FP (n-1) were not significantly larger than those for short FP (n-1), (*t*(16) = 1.58, *d* = 0.29, *n*.*s*.). The mean numbers of trials for short and long FP (n-1) were 17.65 (±4.43) and 14.53 (±3.99).

Then, we calculated the inter-individual correlation between the individual magnitudes of FP/SE effects for RT and M-PUI; cf. Fig 1(E). Our previous results indicated that the trial-by-trial correlation between M-PUI and RT per trial was as high as 0.3. Therefore, we expected that the inter-individual correlation coefficient, if there were a correlation, between the FP/SE effects on M-PUI and RT based on those indices averaged for each individual would be as strong as 0.6~0.8. A Pearson’s correlation coefficient of 0.7 and a sample size of 17 would result in 90% power for rejecting the null hypothesis at .05, which is above the acceptance criterion of 80% [44].

**Fig 6:**
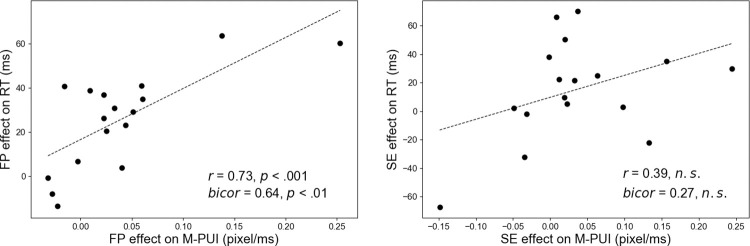
Scatterplots of individual magnitudes of FP/SE effects on RT and M-PUI.

We first calculated the inter-individual correlation between the FP effects on RT and M-PUI; cf. the upper panel of Fig 1(E). As shown in Fig 4(A), the FP effects on RT/M-PUI for each individual were the average RTs/M-PUIs for short FP (n) minus the average RTs/M-PUIs for long FP (n), respectively. We tested for an inter-individual positive correlation between the individual magnitudes of the FP effect on RT and M-PUI using Pearson correlation coefficient (*r*) and Biweight midcorrelation (*bicor*), which is an outlier robust, median-based correlation metric [45]. The scatterplots between the FP effects on M-PUI and RT are shown in the left panel of Fig 6. As expected, there was a significant positive inter-individual correlation between the FP effects on RT and M-PUI (*r* = 0.73, *p*<001; *bicor* = 0.64, *p*<01).

We also calculated the inter-individual correlation between the SE effects on RT and M-PUI; cf. the lower panel of Fig 1(E). As shown in Fig 4(B), the SE effects on RT/M-PUI for each individual were the mean RTs/M-PUIs for long FP (n-1) minus the mean RTs/M-PUIs for short FP (n-1), respectively. The scatterplots between the SE effects on M-PUI and RT are shown in the right panel of Fig 6. There was no significant inter-individual correlation between the SE effects on RT and M-PUI (*r* = 0.39, *n*.*s*.; *bicor* = 0.27, *n*.*s*.).

**Fig 7:**
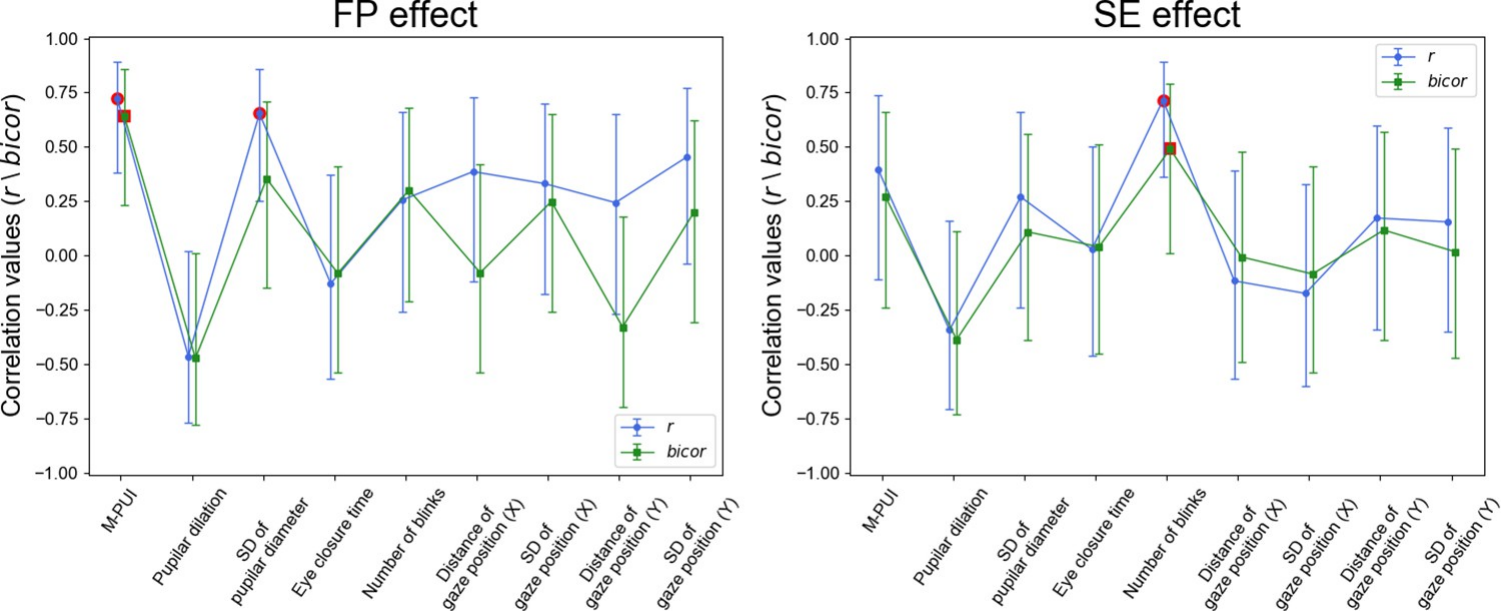
Correlation between FP/SE effects on RT and eye movement indices compared to M-PUI. The red circles indicate that the eye movements reached a significance level of .05. Error bars represent 95% credible intervals.

Finally, we examined the inter-individual correlation between the FP/SE effects on RT and other eye movement indices, including the mean pupilar dilation, standard deviation (SD) of the pupilar diameter, the mean eye closure time, the mean number of blinks, the mean gaze position distance from the center of the screen (in X and Y-axis), and the SD of the gaze position (in X and Y-axis). The inter-individual correlations between the FP/SE effects on RT and eye movement indices are shown in Fig 7. Only the M-PUI was significant for the FP effect, as indicated by Pearson’s correlation coefficient *r* and *bicor*. Moreover, only the number of blinks was significant for the SE effect, as indicated by *r* (*p* < .01) and *bicor* (*p* < .05).

**Fig 8:**
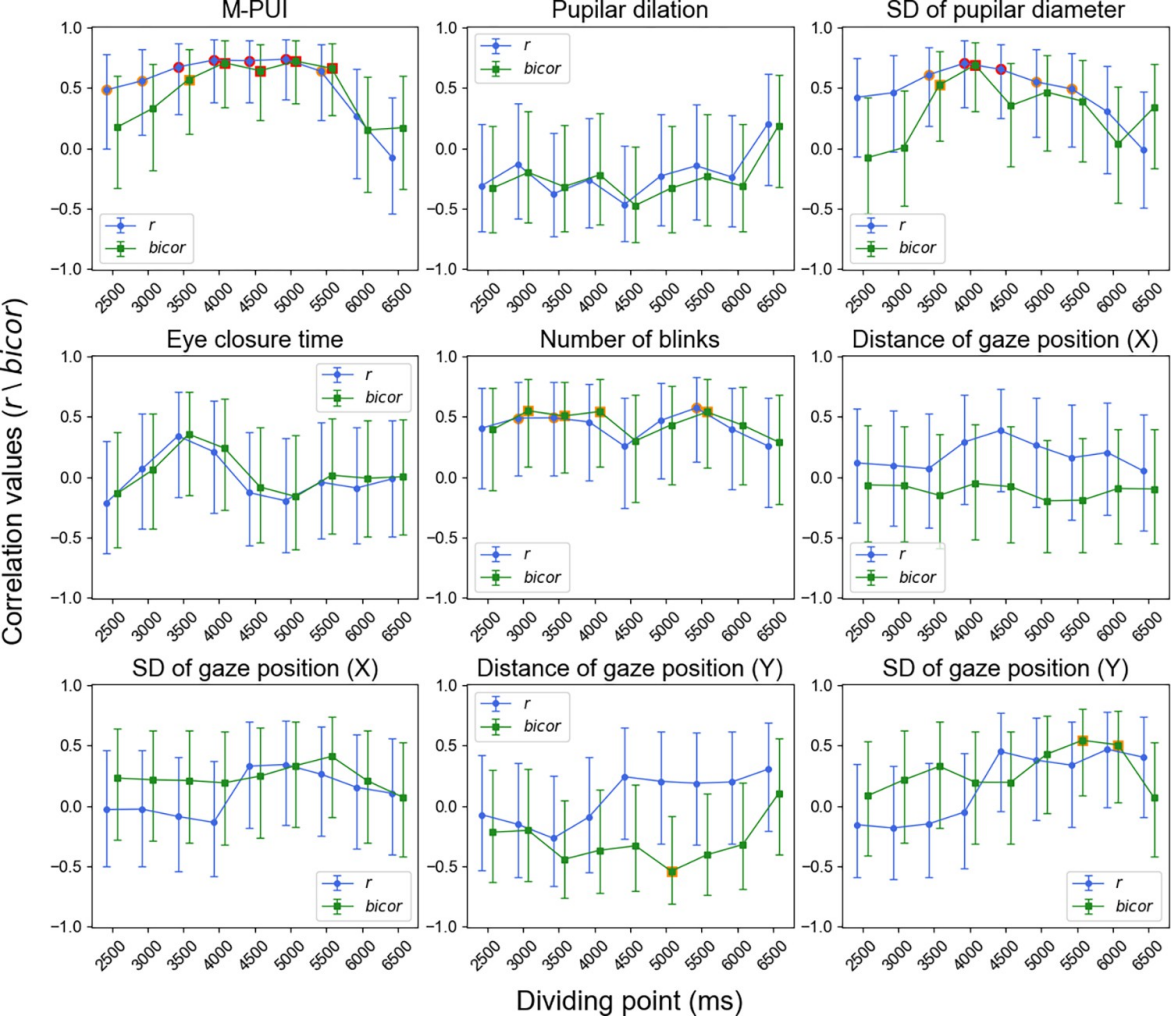
Dividing-point-dependent correlation of FP effect on RT for M-PUI and eye movement indices. The red circles indicate the dividing points that reached a corrected significance level of .0055. The orange circles indicate the dividing points that reached an uncorrected significance level of .05. Error bars represent 95% credible intervals.

We conducted supplementary analyses to test further the robustness of the inter-individual positive correlation between FP/SE effects on RT and M-PUI using different dividing points that ranged from 2,500 to 6,500 ms in 500 ms increments (see the “dividing point” in Fig 4). The correlations between FP effects on RT and all the eye movement indices for each dividing point are shown in Fig 8. The results of Pearson’s correlation coefficient *r* and *bicor* again indicated that only the M-PUI reached a strictly corrected significance level of *p* < .0055 for dividing points of 4,000 to 5,000 ms. Except for the SD of the pupilar diameter, no other eye movement indices showed a significant correlation at a corrected level of *p* < .0055. The mean numbers of trials for short FP (n) in each condition were 14.35 (±5.49), 20.00 (±6.63), 26.06 (±7.14), 32.00 (±7.58), 38.06 (±8.40), 44.53 (±9.11), 51.00 (±9.89), 57.41 (±11.12), and 64.12 (±11.96). The mean numbers of trials for long FP (n) in each condition were 69.24 (±10.10), 63.59 (±9.03), 57.53 (±8.56), 51.59 (±7.92), 45.53 (±7.23), 39.06 (±6.10), 32.58 (±5.39), 26.18 (±4.18), and 19.47 (±3.01).

**Fig 9:**
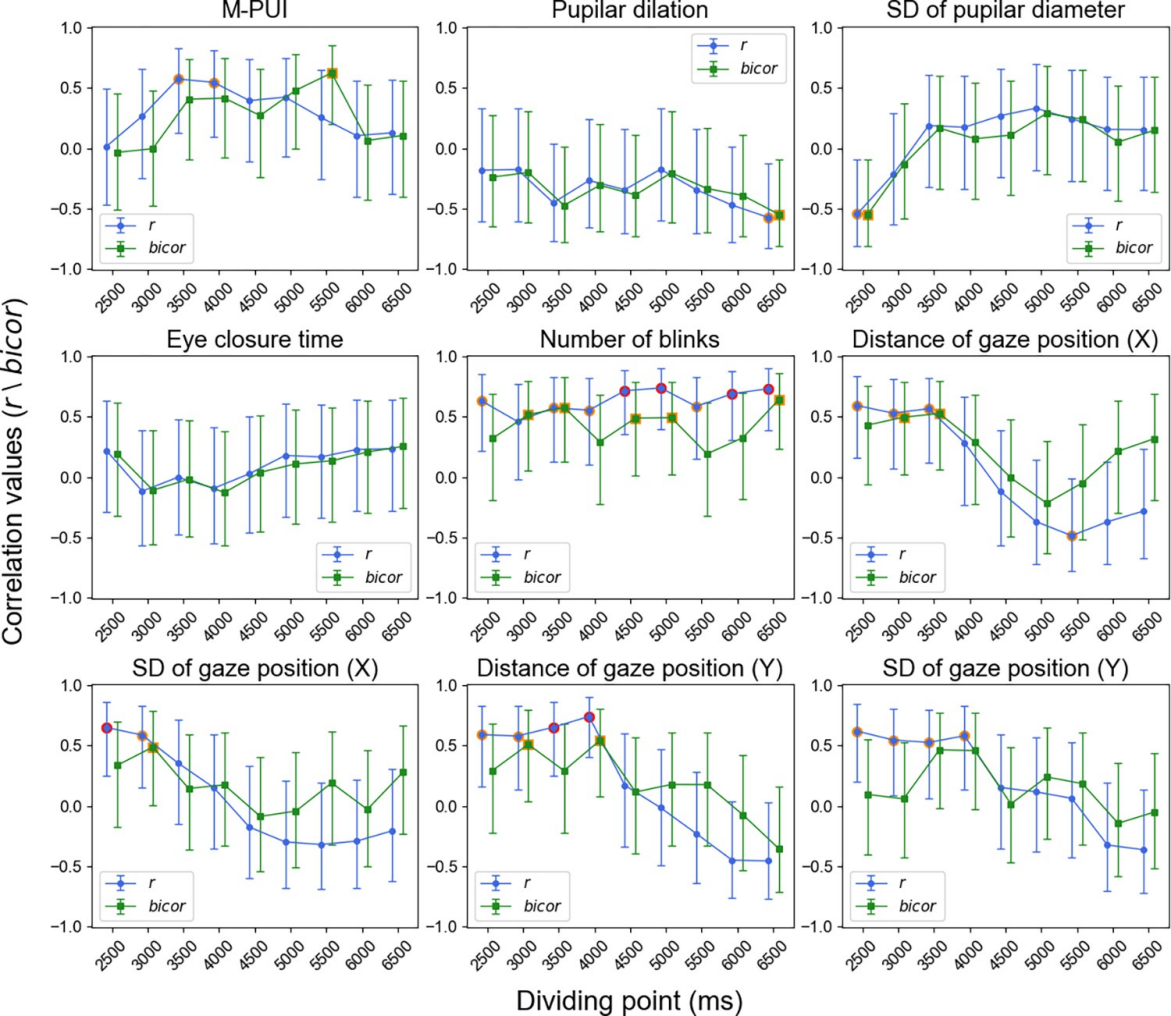
Dividing-point-dependent correlation of SE effect on RT for M-PUI and eye movement indices. The red circles indicate the dividing points that reached a corrected significance level of .0055. The orange circles indicate the dividing points that reached an uncorrected significance level of .05. Error bars represent 95% credible intervals.

The correlations between the SE effects on RT and all eye movement indices for each dividing point are shown in Fig 9. Again, Pearson’s correlation coefficient *r* and *bicor* indicated that the M-PUI did not reach the corrected significance level of *p* < .0055 at any dividing point. Pearson’s correlation coefficient *r* showed that several other indices reached the corrected significance level of *p* < .0055, but the outlier robust *bicor* did not reach significance. The mean numbers of trials for short FP (n-1) in each condition were 7.41 (±2.09), 9.65 (±2.30), 12.41 (±3.40), 14.94 (±3.95), 17.65 (±4.43), 20.06 (±5.20), 22.59 (±5.78), 24.82 (±6.00), and 26.88 (±6.49). The mean numbers of trials for long FP (n-1) in each condition were 24.76 (±6.01), 22.53 (±5.65), 19.76 (±4.85), 17.23 (±4.25), 14.53 (±3.99), 12.12 (±3.18), 9.59 (±2.68), 7.35 (±2.35), and 5.29 (±1.87).

## Discussion

### M-PUI’s validity in estimating temporal attention

The current results suggest that the M-PUI dynamically reflects the FP effect in which temporal attention varies according to the current FP of several seconds. We reproduced the FP effect on RT, in which the RT is shorter when the current FP is longer. Similarly, the M-PUI was shorter when the current FP was longer. In addition, the decrease in M-PUI immediately before target presentation, i.e., 1,000 ms (cf. [35]), with the increasing current FP was positively correlated with that of RT on an inter-individual basis. Therefore, the M-PUI might reflect the degree to which temporal attention increases according to the mechanisms underlying the FP effect (cf. [11–15]).

The M-PUI may be the first step in estimating temporal attention in realistic, medium-term variable FP situations. The FP effect is only one typical effect observed in the simplest of possible temporal attention situations in which a response to a target is always required after the medium-term variable FPs under the uniform distribution. Nevertheless, none of the pupilar indices used to date can estimate temporal attention during the variable FP, even in this most straightforward situation (cf. Table 1). One study reported that the pre-target average pupilar diameter was negatively correlated with the current FP; however, this finding could not be interpreted and was considered noise caused by the task situation [24]. In contrast, the M-PUI robustly reflected differences in the current FP and corresponding differences in RT, suggesting its promise as a temporal attention index in the medium-term variable FP paradigm. However, temporal attention in the real world is more complex and combines different conditions, including the distributions of target appearance, uncertainty about target appearance, and multiple responses to a target. The relationship between M-PUI and temporal attention in complex situations requires future study. If M-PUI passes these tests, it could be used as a research tool for investigating temporal attention and cognition in real-life, medium-term variable FP situations even before explicit responses to targets. M-PUI could also be used for usability evaluation of different systems and for developing adaptive intervention systems based on sensing users’ real-time temporal attention in the medium-term variable FP tasks.

This study also suggested that the M-PUI might not reflect the SE effect that varies according to the previous FP. The current study did reproduce the SE effect for RT by showing that the RT to the target presented in the early part of the current trial was longer if the previous FP were longer. However, the M-PUI was not significantly longer when the previous FP was longer. In addition, the decrease in M-PUI immediately before target presentation, i.e., 1,000 ms (cf. [35]), when the previous FP decreased, was not correlated with RT on an inter-individual basis. Different biological systems might regulate temporal attention in response to the FP and SE effects (cf. [12,32–34]), and the M-PUI may only reflect the system’s state for the FP effect.

The finding that M-PUI might reflect the FP effect but not the SE effect of temporal attention extends our findings on M-PUI. Our previous study found that M-PUI correlated with RT on a within-individual trial-by-trial basis, regardless of the length of current or previous FPs [35]. As a result, factors causing the within-individual trial-by-trial RT variation reflected by the M-PUI were unclear. In contrast, the current study calculated M-PUI for each short/long FP within each individual and indicated that M-PUI based on the current FP reflects within-individual trial-by-trial RT variation only caused by the FP effect. This finding was further supported because the M-PUI only reflected the degree of change in each individual’s RT based on the current FP, which is an issue that has not previously investigated. We had previously indicated that the trial-by-trial correlation between M-PUI and RT did not disappear after excluding the effect of the current FP. The previous finding, together with the current study’s results, suggests that while the M-PUI mainly reflects the FP effect, it also reflects trial-by-trial variation in RT of the same length as the current FP. On the other hand, although we had previously suggested that the latter trial-by-trial variations might also be affected by the previous FP, i.e., SE effect, we withdraw this claim based on the current results.

### M-PUI’s validity in estimating individual differences in temporal attention

The current study might have included different factors that caused inter-individual variability in the FP and SE effects. Medium-term variable FP studies on the FP effect suggest that people conditioned to perform well on the task consistently have shallower FP (n)-RT slopes with generally shorter RTs [21,24,37]. On the other hand, there are no consistent interpretations regarding individual differences in the SE effect [21,38]. For example, firstly, the FP effect generally decreases, showing shorter mean RTs in people with high working memory capacity [37]. Secondly, the FP and SE effects decrease with increasing arousal levels, such as in people with adequate sleep compared sleep deprived people [21]. Thirdly, reward motivation decreases the FP and SE effects [24]. Finally, the SE effect increases in people with low impulsivity [38].

The current study did not control participants’ working memory capacity, sleep schedules, or personality traits, which might have caused individual differences in participants’ temporal attention in the variable FP task. The motivation to perform the task might differ from one individual to another, even though the rewards were not different for different participants. These background factors might affect biological systems controlling temporal attention in the medium-term variable FP task, and the M-PUI could reflect these systems’ conditions. M-PUI’s validity in estimating individual differences is critical because it is essential to account for individual differences in participants’ temporal attention in realistic environments and develop user-friendly systems by estimating temporal attention. One direction for future research is investigating factors causing individual differences in biological mechanisms supporting temporal attention in medium-term variable FP tasks.

### Comparing M-PUI and other eye movement indices

There are no indices of eye movements during the FP that robustly reflect the FP effect on RT other than the M-PUI. The M-PUI cannot be substituted by the widely used pupilar dilation index (i.e., a pre-target average of the baseline-corrected pupilar diameter). The pupilar diameter’s SD might reflect the FP effect on RT; however, the M-PUI appears to extract signals better. The M-PUI focuses only on short-term fluctuations of pupilar diameter (i.e., dilations and contractions smoothed by a temporal resolution of 75 ms in the current study). In contrast, the pupilar diameter’s SD includes all the variability in the pupilar diameter’s time series. These findings suggest that temporal attention involving the FP effect is related to biological systems reflected by pupilar diameter’s short-term fluctuations rather than to the mean pupilar diameter or any other pupilar diameter variability. Also, the effects of short-term pupilar diameter fluctuations cannot be substituted by possible artifacts caused by the eye closure time, the number of eyeblinks, or gaze position variations.

### Possible mechanisms underlying the current results

We have proposed two possible mechanisms underlying the M-PUI’s reflection of the FP effect after considering the three temporal attention models: the dual-process model [12,32–34], the multiple trace theory of temporal preparation [14,15], and the recent view [18,46–49]. One possibility is that M-PUI’s reduction reflects the degree of preparation for target detection/reaction that increases as FP (n) increases through the top-down regulation of LC-NE excitatory phasic activity [50] (i.e., the excitatory LC-NE account). Another possibility is that M-PUI reflects the inhibitory signal of perceptual/response representations that increases rapidly when FP (n) is short and decreases gradually when FP (n) is long (i.e., the inhibitory account). The latter suggestion is supported by recent findings on the relationships between pupillary fluctuations and the real-time power of inhibitory brain waves [51]. These are discussed in detail below.

#### Background: Temporal attention in short-term variable FP paradigm

Two mainstream models explain the FP and SE effects of temporal attention in the short-term variable FP paradigm: the dual-process model (DPM) [12,32–34] and the multiple trace theory of temporal preparation (MTP) [14,15]. Furthermore, recent studies have revealed valid features of the DPM and MTP that we have called the “recent view (RV)” [18,46–49]. We, similar to previous studies [21,23,24], assumed that these models are applicable to the medium-term variable FP task used in the current study for discussing the possible mechanisms of the current results.

#### Background: The dual-process model (DPM)

The DPM assumes that top-down and bottom-up processes produce the FP and SE effects [12,32–34]. The top-down process is assumed to function as a monitor/updater of temporal attention according to the conditional probabilities of target presentations (cf. [11]). Given that a target has not been presented, the conditional probabilities of the target presentation must appear to increase as time passes during a trial. The participants are assumed to increase their temporal attention according to these increasing conditional probabilities when the FP (n) is extended and forms faster responses to a target, resulting in the FP effect.

In addition, the bottom-up process is assumed to cause a trial-by-trial, constant, higher (lower) automatic motoric arousal after a shorter (longer) FP (n-1). However, the top-down effect is assumed to, in some way, overwrite these automatic differences in motoric arousal in longer FP (n). Consequently, RT differs due to FP (n-1) only in shorter FP (n), resulting in the SE effect. Neuropsychological studies have shown a double dissociation in the localization of top-down and bottom up-processes [32–34]. The right prefrontal cortex is involved in the top-down process [32], whereas the left premotor area is associated with the bottom-up process [33].

### Background: The multiple trace theory of temporal preparation (MTP)

The MTP assumes that a response is made according to the sequences of the inhibition/activation reaction’s strength based on the memory traces formed up to a given trial [14,15]. The MTP assumes sequential temporal information learning between the warning signal (i.e., announcing the start of a trial) and the target. Suppose that a participant responds to a target presentation after a specific FP (n) following a warning signal. In this trial, the activation of the response before the FP (n) is low (inhibited) but high (activated) at that FP (n), which leaves a memory trace. As a result, multiple memory traces are left and accumulated in repeated trials. It is assumed that the response tendency is determined based on these traces such that the target would be slower (faster) if the response is inhibited (activated) at a referenced time in multiple memory traces.

The MTP explains the FP effect by considering the biased number of memory traces for FP lengths (see Fig 3 in [14]). The number of memory traces indicating excitation is uniform for each FP when, for example, the target presentation probability is uniform for each FP. On the other hand, the number of memory traces decreases as FP increases. Therefore, dividing the number of excitatory traces by the whole number of memory traces shows a greater proportion of inhibition when FP is shorter and a greater proportion of excitation when FP is longer. Consequently, there might be rapid development and gradual release of inhibitions during the whole FP (n), i.e., a gradual increase of activations during the later time in the FP (n). These processes result in the RTs in a given trial being biased toward being faster as the FP (n) is extended (i.e., the FP effect).

The MTP explains the SE effect based on the identical but especially strong mechanism as the FP effect (see Fig 3 in [14]). Specifically, the memory traces may have a stronger influence the closer they are to a given trial, such that a memory trace in a preceding trial might have a particularly large impact. Consequently, if the FP (n-1) is long, the response to the target presented after the short FP (n) is assumed to be delayed due to inhibition in a preceding, strong memory trace. On the other hand, if the FP (n-1) is short, the response to the target presented after the short FP (n) is assumed to be accelerated due to strong activation. The FP (n-1) effect disappears at longer FP (n) because this process refers only to a longer FP (n-1) time when the weak inhibition has been memorized close to the response activation if there is a preceding referenceable trace, such as in the case of long FP (n-1).

#### Background: The recent view (RV)

Recent studies have supported a modified version of the MTP, where different memory traces have different influences on the FP and SE effects, incorporating the DPM’s physiological suggestions [18,46–49]. These studies have reproduced the dynamically varying RTs according to past experiences, supporting the MTP [46,47]. More precisely, research has suggested that long-term memory traces supporting the FP effect is formed through a process of selective encoding and retrieval of stimulus features related to the task [46,47], whereas the short-term memory trace supporting the SE effect is formed automatically [18,48]. That is, the long-term memory traces supporting the FP effect might be of a different category than short-term memory traces supporting the SE effect [18]. These recent studies have indicated alpha waves’ power as a physiological mechanism, suggesting that the top-down-modulated inhibition signal [52] rapidly increases in shorter FP (n) and decreases as FP (n) lengthens [49], which is consistent with the DPM and the modified version of the MTP.

#### Excitatory LC-NE account of how M-PUI reflects the FP effect

We first propose that the reduction of the M-PUI might reflect the degree of LC-NE’s preparation for the phasic enhancement of target detection/reaction that increases as FP (n) increases. As assumed previously [35], this excitatory LC-NE account considers that the participant’s pupilar diameter, which is unstable in the baseline state, is stable in the preparatory state for LC phasic activity [35]. The LC phasic activity, reflected in the post-target transient pupilar dilation, is thought to facilitate target detection through top-down regulation [50]. Tightly coupled to the LC [27,50], the prefrontal cortex is assumed to function as the top-down process in the DPM, RV, and LC phasic activity. Therefore, the preparation for the LC phasic activity at a certain FPs (n) might be strengthened as the FP (n) lengthens, reflecting the DPM’s conditional probability-based attention or the RV’s activation strength based on long-term memory traces, i.e., the memory traces over two trials before the current trial. Consistent with this idea, the pupilar diameter shows a more significant post-target transient dilation at a certain FP (n) when participants are assumed to attend, compared to other FP (n) [24]. In this situation, the pupillary fluctuation amplitude (i.e., M-PUI) might decrease as the FP (n) lengthens. In addition, participants that can immediately develop temporal attention might generate decreased pre-target pupillary fluctuations and a larger post-target LC phasic activity even in shorter FP (n). Consequently, during the shorter FP (n), when post-target LC phasic activity would occur more strongly only in individuals with an efficient top-down process, the LC may be already prepared for phasic activity before the target presentation, thereby stabling the pre-target short-term fluctuations, i.e., smaller M-PUI. The greater the decrease in the pupillary fluctuation amplitude, even with shorter FP (n), the lesser might be the reduction in M-PUI and RT with increasing FP (n), resulting in the inter-individual correlation. Such changes might appear mainly in the pupillary fluctuation amplitude at a temporal resolution of 10–20 Hz, which corresponds to the phasic activity frequency range of the LC [35] rather than the mean pupilar diameter.

Below, we have summarized the relationship between the contemporary LC-NE excitatory account and previous temporal attention models. It is reasonable to assume that M-PUI reflects the FP but not the SE effect in terms of the DPM and RV, which suggest separate processes in the FP and SE effects. In contrast, it is inconsistent that M-PUI’s selective reflection of the FP in terms of the MTP, which emphasizes long-term (over two trials before the current trial) and short-term (one trial before the current trial) memory traces, operates through the identical unidimensional inhibition/activation mechanism [14]. Perhaps we must include the assumptions that functionally unidimensional but physiologically different mechanisms support the FP and SE effect separately from the MTP. That is, there might be a physical site for long-term memory traces that correlates with M-PUI and another site for short-term memory traces that do not correlate with M-PUI.

One difficulty with the above scenario of the medium-term variable FP paradigm is a study suggesting that post-target transient pupilar dilation does not increase as the FP (n) lengthens [24]. This study indicated that participants with reward motivation develop compensatory attention at shorter FP (n), which results in increased LC phasic activity at that time [24]. However, the pupilar diameter does not always perfectly capture LC activity because the assumed LC activity might be observed in the M-PUI but not in post-target transient pupilar dilation [53]. Nevertheless, it is desirable to develop a more plausible explanation that accounts for inhibition, which is not included in the excitatory LC-NE account, although the LC-based account has often been assumed in traditional pupilar indices [50].

#### Inhibitory account of how M-PUI reflects the FP effect

We also propose that M-PUI might reflect the degree of perceptual/response inhibition representations that rapidly increase when FP (n) is short and decreases gradually when FP (n) gets longer. The inhibitory account considers that participants’ pupilar diameter is stable in the baseline condition but unstable in the inhibited state (cf. [51]). The increased inhibitory signals in the MTP and RV might be observed when target presentation is improbable under decreased temporal attention at shorter FP (n). A recent body of findings suggests that pupillary fluctuations (e.g., the first derivative of the pupilar diameter) reflect the global state of the central nervous system rather than solely the specific neural activity in the LC-NE [53–56]. A notable recent study has shown pupilar diameter fluctuations with periods as high as 1 Hz vary in seconds, reflecting the power of alpha waves, suggesting the top-down-modulated inhibition signal [52] in a fixation task [51]. These features seem to fit well with the M-PUI, which is calculated from the summation of the pupilar diameter time series differentials on a temporal resolution of 10–20 Hz. If this study’s task concerned the relationship between inhibitory alpha power and the pupilar diameter (cf. [51]), then the M-PUI might capture the minute pupilar dilations and constrictions, reflecting the rapid development and gradual release of inhibitory alpha during the time course of the whole FP (n), which is generated by the long-term memory traces. The power of inhibitory alpha and M-PUI may be larger for shorter FP(n) but smaller for longer FP (n). Furthermore, the greater the inhibition, e.g., the power of inhibitory alpha and M-PUI, with decreasing FP (n), the greater the increase in RT with decreasing FP (n), resulting in inter-individual correlations.

Below, we have summarized the relationship between the inhibitory account and previous temporal attention models. The DPM does not suggest this account because it does not mention inhibitory mechanisms; however, there is no contradiction between this account and the DPM. The MTP suggests this account because it provides an inhibitory account of the FP and SE effects. However, it is nevertheless inconsistent with the selective reflection of the FP effect by the M-PUI. Similar to the excitatory LC-NE account, we have assumed that physiologically diverse mechanisms separately support the FP and SE effect in the MTP. The RV is highly consistent with this account because M-PUI is related to the top-down, inhibitory FP effect but not to the bottom-up SE effect.

#### Limitations and future theoretical directions

For the time being, the above discussion remains a hypothesis worth investigating. Future research is needed to clarify whether the results of the current study can be explained by excitation, inhibition, or both mechanisms. Furthermore, the specific physiological pathways by which M-PUI might capture inhibition-related signals remain unclear. We suggest that it might occur through the interplay (cf. [51,55,57]) between the LC-NE [50] and the cholinergic system [55,57].

A possible direction for future research might be to consider the quick dynamic acquisition of the internal probability estimates in the past few trials, which results in executing perceptual inputs and motor outputs in the current trial (cf. [58]). The combination of the M-PUI’s reflection of current temporal attention and top-down control associated with modifying internal (subjective) probability estimates in past trials might explain the current results through the excitatory LC-NE account, yet the inhibition of representations in the current trial (i.e., the inhibitory account) could also explain those. It might be necessary to investigate the nature of long-term memory traces (i.e., those over two trials before the current trial), which were only roughly defined in the current study, in more detail. Note that, since our previous study confirmed a trial-by-trial relationship between M-PUI and RT [35], it is unlikely that the M-PUI reflects only the factors related to the acquisition stage of target presentation distributions in past trials.

### Current study’s limitations and future directions

The findings of the current study are constrained by several limitations. Firstly, the range of variable FPs used in the current study was limited. Many typical variable FP paradigms, which we call the short-term variable FP paradigm (see the Introduction), have been investigated at very fine temporal resolutions, with FPs ranging from less than 1 s to at most 2 s [16–18]. On the other hand, the current study focused on the medium-term variable FP paradigm [20–24] and examined FP and SE effects using variable FPs of 1–8 s, which is typical for PVT [19,41]. Many previous studies of the medium-term variable FP in PVT have confirmed the FP and SE effects [20–24]. However, the typical short-term variable FP paradigm and the medium-term FP paradigm in PVT might have very different mechanisms. For example, it has been shown that the degree of SE effects varies depending on the range of FPs [59]. Although the current results are primarily on top-down FP effects, the differences in SE effects might not be negligible because they might include arousal-related effects that might be associated with top-down effects [60]. Alternatively, they might differ because the effect sizes are smaller in the short-term variable FP paradigm due to the time taken by the pupil to reflect cortical activity [55]. Therefore, the current findings and hypotheses need to be tested using a broader range of FPs, including more short-term FPs.

Secondly, there are other differences between the experimental manipulation of a typical variable FP paradigm and the current task. This study followed the original PVT in investigating the effect of variable FP in the PVT. Consequently, we terminated the target presentation immediately after a response. We also presented RT as feedback. Furthermore, we did not judge a miss until 60 seconds after the target presentation [19]. These experimental procedures might have caused differences between the current study and studies using the typical variable FP paradigm. We describe these settings to indicate the scope of generalizable experimental settings because it is well-known that seemingly trivial manipulations of the experimental procedures can influence results (cf. [61]).

Thirdly, the findings of this study are limited because of reusing old data. From the perspective of the experimental manipulation, we verified the current findings only in one specific experimental situation. Therefore, it might be necessary to verify the robustness of the current findings by changing the parameters of the current experimental situation. From the perspective of statistical hypothesis testing, each repeated independent hypothesis test for a given data set leads to an increase in the probability of rejecting the null hypothesis [62]. Although, previous studies have indicated that strictly correcting for the significance level is undesirable because it would inhibit meaningful findings [62]. Nevertheless, the increased risk of rejecting the null hypothesis remains a limitation of the current study.

## Conclusion

The M-PUI before target presentation reflected the variable foreperiod effect (i.e., the FP effect) on RT and even reflected individual differences in the FP effect on RT in the medium-term variable FP paradigm. Also, the M-PUI can be calculated before presenting a target. Therefore, it has the potential as a tool for estimating attentional fluctuations in the applied contexts. We have discussed the possible association between this finding and the phasic activity of the LC and the inhibitory signals as a mechanism underlying this effect.
